# A neonate with marked prolonged mixed apneas and CHARGE syndrome: a case report

**DOI:** 10.1186/s13256-018-1748-2

**Published:** 2018-09-04

**Authors:** M. A. Riedijk, A. M. König-Jung, J. B. M. van Woensel, F. H. Kruisinga

**Affiliations:** 1 Department of Pediatric Intensive Care, Emma Children’s Hospital Amsterdam UMC, PO Box 22700, 1100 DE Amsterdam, Netherlands; 2 Department of ENT, Amsterdam UMC, Amsterdam, the Netherlands; 3 Department of Pediatrics, Emma Children’s Hospital Amsterdam UMC, Amsterdam, the Netherlands

**Keywords:** Mixed apneas, Obstructive breathing, Polysomnography, Tracheostomy, Infant

## Abstract

**Background:**

Upper airway abnormalities in the newborn are associated with obstructive breathing but not with mixed apneas. A tracheostomy is necessary to treat severe obstructive apneas but will not have an effect on the central part of the mixed apneas. As far as we know, this is the first case report describing disappearance of severe mixed apneas after tracheostomy in a 7-week-old infant.

**Case presentation:**

We report a case of a white female neonate with anatomical upper airway abnormalities and severe mixed apneas with desaturations needing respiratory support. Polysomnography revealed striking mixed apneas, starting as a prolonged central apnea and merging into an obstructive apnea, and were not appropriate for her age. Additional examination revealed no explanation for the central component of the mixed apneas. Because of persistent, severe desaturations, she needed respiratory support with failure to wean. Finally, a tracheostomy was performed to treat the obstructive apneas, but unexpectedly the central apneas also resolved. Recently, additional genetic testing revealed that she has CHARGE syndrome (coloboma of the eye, heart defects, atresia of the choanae, retardation of growth and development, genital and/or urinary abnormalities, and ear abnormalities and deafness).

**Conclusions:**

Mixed apneas are not a common feature in the newborn or infant with upper airway abnormalities. However, treatment with tracheostomy in our patient (day 46 postpartum) with anatomical upper airway abnormalities resolved not only obstructive apneas but also, unexpectedly, severe mixed apneas. Surprisingly, a posttracheostomy polygraph showed only short central apneas appropriate for age.

## Background

There are three types of sleep apneas: obstructive, central, and mixed. Obstructive breathing is often associated with upper airway abnormalities. Polysomnography (PSG) is the gold standard for diagnosing these so-called obstructive sleep apneas (OSAs). Severity of OSA is expressed as obstructive apnea-hypopnea index (OAHI) in episodes per hour [1]. In general, infants have a different sleeping pattern than older children, and central apneas are common during rapid eye movement sleep [2]. In particular, marked central apneas are frequently seen in preterm neonates and attributed to central irregularity [3]. However, upper airway abnormalities are related to OSA, although a combination with mixed apneas can occur. If severe upper airway obstruction is present, a tracheostomy could be considered, though this will have an effect on obstructive apneas but not central apneas.

## Case presentation

A 1-day-old white female newborn was admitted to the pediatric intensive care unit of our hospital because of persistent severe obstructive respiration with cyanosis of unknown etiology. She had been born in a peripheral hospital with a gestational age (GA) of 39 weeks, 1 day, with a birthweight of 2790 g and Apgar scores of 5 and 7 at 1 and 5 minutes, respectively. Pregnancy was uneventful with normal routine prenatal ultrasounds. Directly after birth, the girl showed apneas with profound desaturations. Respiratory support with continuous positive airway pressure (CPAP) was started without improvement. Also, antibiotic treatment was started for a neonatal infection. On admission, her central temperature was 38.2 °C, her heart rate was 145 beats per minute, her blood pressure was 65/40 mmHg, her oxygen saturation level was > 99% on room air and nasal CPAP, and her positive end-expiratory pressure (PEEP) was 3 cmH_2_O. On physical examination, she had a few dysmorphic features: small fontanelle, downward slanting eyes, low-set left ear, retrognathia, and sandal gap of both feet. Her heart rate was regular with normal heart sounds. Her voice was hoarse, and with CPAP she did not have an inspiratory stridor and showed no signs of increased work of breathing, with normal air entry of both lungs without rhonchi or crackles. Her neurological evaluation revealed no abnormalities. We considered the following in the differential diagnosis: upper airway disorder, congenital heart disorder with spells, intoxication, infection, neurologic etiology, and central hypoventilation syndrome.

Additional examinations were performed. Her blood test showed normocapnia with balanced electrolytes and no signs of infection. Her urine toxicology test did not show any intoxication with amphetamines, benzodiazepines, methadone, or opioids. Her cardiac ultrasound showed a structural normal heart with no signs of pulmonary hypertension, and an ultrasound of her brain did not reveal any abnormalities, and cerebral function monitoring showed no epileptic activity. Because of the dysmorphic features, genetic testing was performed, which showed a normal DNA profile with 46 chromosomes.

On day 2, a flexible bronchoscopy was performed, which demonstrated large arytenoids with relatively short vocal cords with sufficient residual lumen of the trachea. Gastroesophageal reflux therapy was initiated with esomeprazole, with no effect. Weaning from CPAP failed because obstructive apneas with profound desaturations returned. Subsequently, a rigid bronchoscopy was performed and demonstrated ongoing obstruction of the laryngeal airway by enlarged arytenoids and still short vocal cords in combination with an anatomic small larynx but without subglottic, tracheal, or main bronchus abnormalities. These findings in combination with retrognathia explained the obstructive breathing.

In the following period, weaning from CPAP failed, and respiratory support needed to be intensified to noninvasive ventilation (PEEP 5 cmH_2_O, pressure support 12 cmH_2_O, respiration rate [RR] 30 breaths per minute). A second flexible bronchoscopy (day 23) was performed to exclude an upper airway obstruction. It showed findings similar to those before. Whether this contributed to the obstructive apneas could not be excluded, because the patient was examined in upright position and not in supine position. No laryngeal and subglottic abnormalities were seen on a magnetic resonance imaging (MRI) study of the mouth and mandible.

**Fig. 1:**
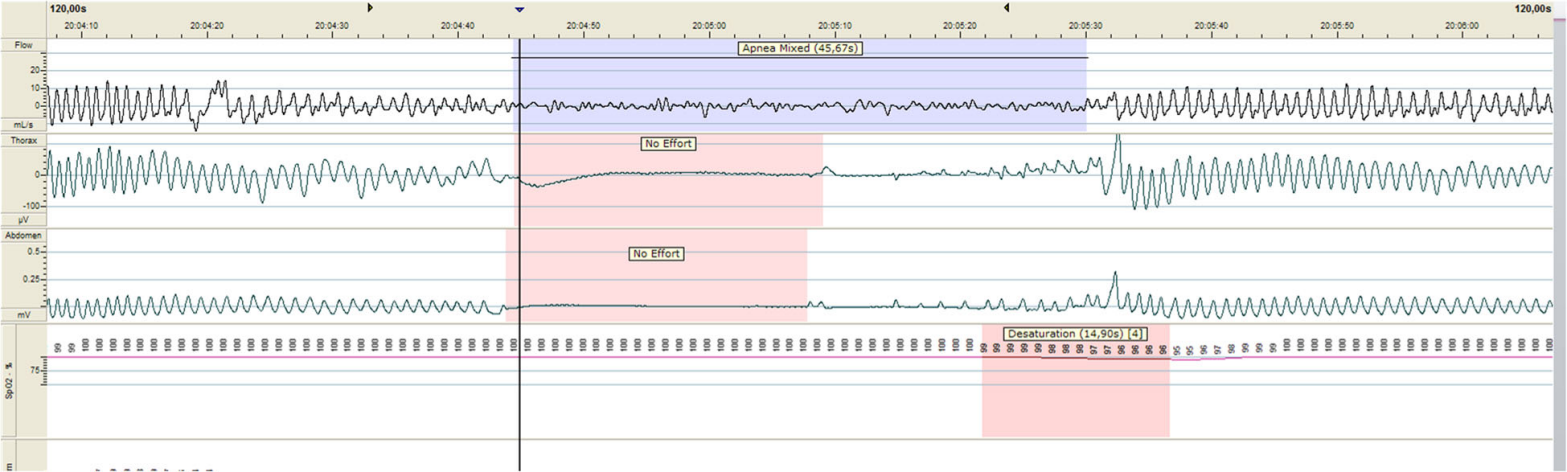
A 2-minute polysomnographic epoch during sleep showing a mixed apnea, starting as a long central apnea

**Fig. 2:**
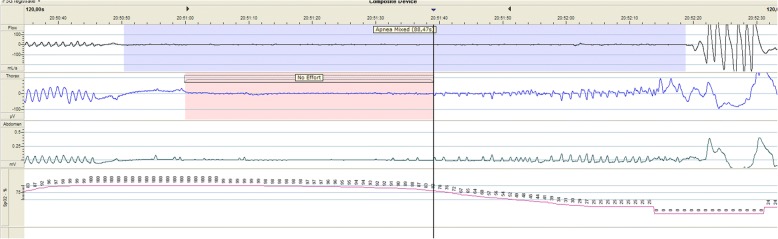
A 2-minute epoch showing a prolonged mixed apnea followed by a profound desaturation

Besides the obstructive apneas, the patient also increasingly developed central apneas without any signs of an infection. She was treated once with caffeine (loading dose) with no effect, and therefore maintenance was not continued. PSG was performed using American Academy of Sleep Medicine scoring criteria to assess the degree and duration of central apneas and the ratio of central to obstructive apneas [4]. PSG registration (15 hours) including electroencephalography (EEG) was performed on day 39 (GA 44 weeks), which showed both obstructive as well as mixed apneas and some isolated central apneas (central apnea index [CAI] 0.1/hour). The mixed apneas were most prominent and started mostly as a central apnea (length up to 40.6 seconds) and merged into an obstructive apnea (Figs. 1 and 2) leading to severe desaturation. The mixed apnea index was 5 per hour with an average length of 34.7 seconds (8.2–88.5 seconds). Furthermore, the patient’s obstructive apnea index was 4.1 per hour. OAHI without mixed apneas was 5.7 episodes per hour, and total apnea-hypopnea index (AHI) was 10.7 episodes per hour. Mean blood oxygen saturation (SpO_2_) was 98.4%, average desaturation was 10.3%, and lowest SpO_2_ was 30%. The percentage of time spent with SpO_2_ < 90% was 2.7% (22.7 minutes). Additionally, continuous transcutaneous CO_2_ (TCCO_2_) was recorded, which revealed mean TCCO_2_ of 53.6 mmHg with a highest value of 68.6 mmHg. A capillary blood gas investigation revealed pH 7.36, partial pressure of carbon dioxide 56.3 mmHg, bicarbonate level of 31.1 mmol/L, and base excess 4 mmol/L. The results of the TCCO_2_ recording and capillary blood gas analysis confirmed the presence of significant chronic hypoventilation. EEG registration showed reactive rhythmic changes preceding and during a desaturation but without any epileptic activity.

We concluded that our patient had moderate OSA based on OAHI, but when the number and severity of mixed apneas were taken into account, we determined that she had severe OSA with an AHI > 10 episodes per hour. Moreover, central apneas were, in general, part of the prolonged mixed apneas, starting as a central apnea and merging into an obstructive apnea. On one hand, the number and duration of isolated central apneas were within the normal range for the patient’s age. On the other hand, the number of mixed apneas and their length were abnormal for her age, especially the long central apnea preceding the obstructive apnea. To rule out cerebral pathology as a cause of central apneas, MRI of the brain was performed, which showed no signs of cerebral abnormalities. Also, central hypoventilation syndrome was unlikely because PSG confirmed mixed apneas and the patient had shown symptoms of obstructive instead of central apneas since birth.

The prolonged mixed apneas caused profound desaturations associated with severe desaturations and bradycardia. Therefore, our patient needed respiratory support (CPAP PEEP 5 cmH_2_O, pressure support 6 cmH_2_O, RR 15 breaths per minute) and repeatedly failed to wean. Without having an explanation for the prolonged central apneas, we finally decided to treat the girl with a tracheostomy. On day 46, tracheostomy was performed, and the procedure was noncomplicated. A tracheostomy tube (Bivona; Smiths Medical, Ashford, UK) with an internal diameter of 3.5 mm was inserted.

After surgery, no more obstructive or central apneas were observed clinically. A poly(somno)graphy (without EEG) was performed 4 weeks posttracheostomy and revealed only short central apneas (average length 6.5 seconds, longest 11.1 seconds) appropriate for her age. No more mixed or obstructive apneas and no profound desaturations were seen (AHI 3.4 episodes per hour, CAI 2.1 episodes per hour, average saturation 98.4%, oxygen desaturation index 9.1 episodes per hour). She was rapidly weaned from the ventilator and transferred to the pediatric ward.

Over a period of time, the patient’s auricular anomalies became more prominent, and she was diagnosed with deafness. In combination with dysphagia and developmental delay, CHARGE syndrome (coloboma of the eye, heart defects, atresia of the choanae, retardation of growth and development, genital and/or urinary abnormalities, and ear abnormalities and deafness) was considered. Whole exome sequencing (WES) was performed again, and coding regions of *CHD7* (chromodomain-helicase-DNA-binding protein 7) were screened for mutations, which showed a *de novo* mutation deep in the intron (c.5405-17G>A), which confirmed the diagnosis of CHARGE syndrome [5].

## Discussion

We report a case of a female neonate with retrognathia, an anatomical small larynx, and large arytenoids with relatively short vocal cords. She was diagnosed with severe OSA consisting of mixed apneas with persistent necessity of respiratory support and failure to wean. In the mixed apneas, duration of the central apnea preceding the obstructive apnea was strikingly long and not appropriate for her age (GA 44 weeks). We could not find any similar published cases to determine further treatment and prognosis. Mixed apneas are considered as obstructive events and determine, along with obstructive apneas and hypopneas, the OAHI. In our patient, the duration of the central apnea preceding the obstructive apnea was unusually long and not appropriate for her age. Therefore, we discussed whether the very long mixed apnea should be considered as obstructive or as a result of central breathing irregularity in an infant with a preexisting small airway whereby a central apnea caused muscle weakness and subsequent collapse of the airway, resulting in an obstructive apnea. If mixed apneas were taken into account, our patient had an AHI of > 10 episodes per hour, defined as severe OSA syndrome. She needed respiratory support because of the long duration of central components in the mixed apneas with profound desaturations, which did not improve in the following weeks. If the central apneas were a result of an immature breathing pattern, we would have expected this to resolve spontaneously with age.

Without diagnostic evidence for cerebral pathology, we discussed the indication for tracheostomy with our team. A tracheostomy would provide an open airway distally from the obstruction, resolving the obstructive apneas, but with no effect on the central apneas. We also discussed the impact and consequences of a tracheostomy for the patient but also for the parents in their daily lives. An expert ear, nose, and throat team estimated that the girl would need the tracheostomy for 4 to 5 years. Next, we discussed our considerations with the parents. After we provided them with detailed information, they decided to agree with performing a tracheostomy.

Although it was not expected that a tracheotomy would have any effect on the central apneas, it was placed to treat the obstructive apneas. Interestingly, after tracheostomy was performed, the apneas and desaturations clinically disappeared. A posttracheostomy poly(somno)graphy (without EEG) revealed only short central apneas appropriate for age and no more mixed or obstructive apneas. We do not have a clear explanation for this. We expected the obstructive apnea to disappear, but not the central component of the mixed apnea. A possible explanation is that the mixed apneas would also have disappeared without tracheostomy, appropriate for her age postpartum. However, we would have expected to see a decrease in mixed apneas already before tracheostomy was performed, but this was not the case. The tracheostomy tube itself might influence the respiratory center by stimulation or irritation. However, we are not aware of this hypothesis and do not find it a good explanation. However, we have no other explanation to interpret the disappearance of the central apneas after tracheostomy.

## Conclusions

We describe a case of a 7-week-old female infant with anatomical upper airway abnormalities and strikingly severe mixed apneas that we poorly understood. Owing to the severity of her mixed apneas, our patient required airway intervention with tracheostomy, which resulted in complete disappearance of her central apneas.
